# A High-Precision Real-Time Temperature Acquisition Method Based on Magnetic Nanoparticles

**DOI:** 10.3390/s24237716

**Published:** 2024-12-02

**Authors:** Yuchang Zhu, Li Ke, Yijing Wei, Xiao Zheng

**Affiliations:** 1 School of Electrical Engineering, Shenyang University of Technology, Shenyang 110870, China; yuchangzhu1998@sina.cn (Y.Z.); keli@sut.edu.cn (L.K.); yijingwei1999@163.com (Y.W.); 2 CAS State Key Laboratory of Forest and Management, Institute of Applied Ecology, Chinese Academy of Sciences, Shenyang 110016, China

**Keywords:** magnetic nanoparticle, magnetothermal equation, temperature inversion, harmonic amplitude

## Abstract

The unique magnetothermal properties of magnetic nanoparticles enable the development of a high-precision, real-time, noninvasive temperature measurement method with significant potential in the biomedical field. Based on a low-frequency alternating magnetic field excitation model, we construct two additional magnetic field excitation models—alternating current–direct current superposition and dual-frequency superposition—to extract harmonic amplitude components from the magnetization response. To increase the accuracy of harmonic information acquisition, the effects of the truncation error, excitation magnetic field frequency, and amplitude are thoroughly analyzed, and optimal parameter values are selected to minimize the error. A single algorithm is designed for temperature inversion, and a joint algorithm is proposed to optimize the performance of the single algorithm. Under low-frequency alternating-current magnetic field excitation, the autonomous group particle swarm optimization method achieves superior real-time performance in terms of temperature inversion and running time. Compared with the opposition learning gray wolf optimizer and particle swarm optimization–gray wolf optimization, the proposed method achieves reductions of 52% and 68%, respectively. Additionally, under dual-frequency superimposed magnetic field excitation, a higher temperature inversion accuracy is achieved compared with that of the particle swarm optimization–gray wolf optimization algorithm, reducing the error from 0.237 K to 0.094 K.

## 1. Introduction

Acquiring noninvasive temperature data, particularly oncological thermomagnetic data, is critical in the biomedical field [1]. Tumor thermomagnetism, also known as cancer thermotherapy or magnetic thermotherapy, is a novel cancer treatment that destroys cancer cells directly by applying high temperatures, ultimately leading to cell death [2]. In contrast to traditional methods such as chemotherapy and radiotherapy, thermotherapy is a physical treatment that is not limited by the accumulation of toxic side effects [3]. Research has demonstrated that during magnetic thermotherapy, maintaining a local tissue temperature in the range of 40 °C to 43 °C induces necrosis in cancer cells while temporarily inactivating normal cells. Once the temperature is reduced to normal levels, the normal cells are reactivated [4,5]. In addition to anticipated tumor cell death, thermotherapy has been shown to induce unexpected biological responses, such as tumor-specific immune reactions triggered by the expression of heat shock proteins. This highlights the ability of thermotherapy to not only destroy local tumors exposed to heat, but also target distant tumors [6]. The relevant effects of thermotherapy have already been demonstrated in mice; however, for practical application in clinical settings, precise acquisition of the corresponding temperature information is necessary [7]. Thus, precise temperature monitoring of the cancerous area is crucial for maximizing the efficacy of magnetic thermotherapy and minimizing damage to normal cells [8,9,10]. Additionally, the duration of treatment is a key factor in the success of magnetic thermotherapy [11,12]. Therefore, developing a noninvasive temperature measurement technology with high accuracy and real-time capabilities is essential for advancing the field of magnetic hyperthermia and improving its clinical applications.

Currently, various temperature measurement techniques are available for micro- and nanoscales. Most nanothermometers operate based on changes in the intrinsic properties of the nanoprobes as the temperature changes, enabling accurate temperature measurements. Toyli and Neumann [13,14] explored the use of nanodiamonds (NDs) as thermal sensors, which utilize temperature-dependent changes in transition frequencies for precise temperature measurement. However, this method is primarily effective at approximately room temperature, limiting its broader applicability. Lu [15] proposed a novel fluorescent nanothermometer that uses butter as a substrate and an aggregation-induced emission (AIE) dye as the detector. This nanothermometer exhibits decreasing fluorescence intensity and a shorter lifetime with increasing temperature within the physiological range of 27–60 °C, although its effectiveness within this range remains unverified. Li [16] introduced a new method for noncontact, nanoscale temperature detection that achieves high sensitivity and rapid responses through synergistic photoluminescence from various activators by selecting ions with specific degrees of crystallinity. However, their study did not address the feasibility or potential toxicity of these materials in complex environments or biological systems. Quantum dots (QDs) are small semiconductor nanocrystals, typically ranging in size from a few nanometers to tens of nanometers, that can also be utilized as tools for temperature measurements in micro- and nanoenvironments [17]. Maestro [18] demonstrated the ability of QDs to perform temperature measurements by using them as fluorescent nanothermometers under a photon microscope, but the stability of QDs under different temperature conditions is unknown. Albahrani [19] proposed an in situ hybridization technique using QDs for temperature measurement in micro- and nanoliquid films. However, the paper noted significant uncertainty in the pressure measurements, which limits the accuracy of the temperature readings.

Pankhurst [20] details the physical principles of magnetic nanoparticles in biomedical applications, offering a theoretical foundation for further research and their application in the biomedical field. Building on the promising advancements in magnetic nanoparticles, several potential applications have emerged from the current state of the art, including drug delivery and thermometry [21]. At the application level of the thermometers, magnetic nanoparticle thermometers are magnetically transparent and highly stable compared to other types of thermometers, allowing them to accurately measure temperatures in high-temperature environments [22]. Dartmouth Medical Center in the United States pioneered the use of magnetic nanoparticles as temperature measurement tools, leveraging their temperature-sensitive properties, which marked a significant advancement in the field [23]. Reeves [24] determined the relationship between the magnetization strength and temperature of magnetic nanoparticles. Zhao [25] successfully prepared samarium-rich nanoparticles via a thermal decomposition technique, and reported that the nanoparticles exhibited a strong temperature dependence. Yan [26] designed and assembled a comprehensive nanoprobe temperature sensor by synthesizing europium-based metal nanoparticles with organic frameworks, enabling real-time temperature feedback to be obtained by monitoring the thermal response fluorescence emission ratio and the fluorescence lifetime of europium. Qu [27] prepared chitosan nanoparticles with a core‒shell structure via a crosslinking method. These nanoparticles are superparamagnetic and show great potential for temperature information detection. Liu [28] developed a magnetic thermometer based on the principle of paramagnetic resonance using a structure that combines magnetic nanoparticles with nitrogen-vacancy color centers. Bui [29] proposed magnetic nanoparticle thermometry based on magnetic particle spectroscopy as a method for the in situ thermometry of three-dimensional objects. Luo [30] proposed a highly sensitive nanothermometer based on silicon carbide double vacancies, utilizing spectroscopic techniques such as nuclear magnetic resonance and electron spin resonance. Shi [31] suggested that the harmonic spectrum of the magnetization response of magnetic nanoparticles under magnetic-field excitation can characterize the microenvironmental properties of the nanoparticles. By adjusting the amplitude or frequency of the excitation field, changes in temperature and relaxation time can be measured. Zhong [32,33] conducted an in-depth study of the magnetization response signals of magnetic nanoparticles, establishing a model of the relationship between the magnetization signal and temperature under direct current (DC), low-frequency alternating current (AC), and triangular-wave excitations. Zhang [34] introduced magnetic nanoparticles into nuclear magnetic resonance (NMR) temperature measurements, and established a temperature measurement model based on the transverse relaxation rate of NMR. Wang [35] showed via a detailed analysis that the magnetization response of magnetic nanoparticles under dual-frequency excitation greatly improves the accuracy of temperature acquisition.

Although significant breakthroughs have been made in magnetic nanoparticle thermometers, several challenges remain in terms of the accuracy of temperature measurements. The primary reasons are as follows: First, magnetic nanoparticles exhibit different magnetization responses under varying external magnetic fields, which could affect the accuracy of the temperature data. Second, multiple interference factors arise during the harmonic amplitude extraction process. Third, different inversion methods used in temperature retrieval result in variations in accuracy and real-time performance. Given the challenge of balancing temperature control accuracy with time optimization in current research, we designed three different excitation scenarios. We then conducted an in-depth analysis of the magnetization response characteristics of nanoparticles under these varying magnetic field conditions. The core objective of this approach is to enable the flexible selection of the most suitable magnetic field conditions and corresponding inversion algorithms based on specific accuracy requirements. This allows for the dual optimization of both temperature control and time efficiency. In light of these challenges in acquiring precise temperature information from magnetic nanoparticles, the main research focuses of this paper are as follows:

(1) An AC–DC superimposed magnetic field excitation model and a dual-frequency superimposed magnetic field excitation model are developed. These models aim to obtain rich harmonic information under different excitation conditions, thereby increasing the accuracy of temperature inversion;

(2) The accuracy of the harmonic values is increased by analyzing and selecting the optimal values for three key factors—the truncation error, excitation magnetic field frequency, and excitation magnetic field amplitude;

(3) Temperature information is extracted via single-algorithm and joint-algorithm temperature inversion methods, which balance the accuracy and real-time performance of the temperature measurements during inversion.

In this work, we present a detailed explanation of a temperature measurement method based on the magnetothermal properties of magnetic nanoparticles. This method offers a noninvasive technique for acquiring temperature information in micro- and nanoenvironments. We present a theory for the magnetization response of magnetic nanoparticles. Using the low-frequency AC excitation of an external magnetic field, we develop two models: AC–DC superimposed magnetic field excitation and dual-frequency superimposed magnetic field excitation. Since temperature information cannot be obtained directly, we target high-precision harmonic information as an indirect measure, and then retrieve the temperature information by inverting the harmonic amplitude data from the magnetization response of the particles. When high-precision harmonic amplitude data are acquired, relevant optimizations and error corrections are applied. Various inversion algorithms are employed to perform temperature inversion and obtain temperature data. Ultimately, we identify a method and model that achieve low-temperature error and high real-time performance, demonstrating that this approach can be effectively applied for the rapid and precise acquisition of temperature information in micro- and nanoenvironments, particularly in the biomedical field.

## 2. Theory and Modeling

The magnetization response of superparamagnetic iron oxide nanoparticles (SPIONs) under an applied magnetic field is described by the Langevin equation [36,37],

(1) $$M(t)=cm_{s}\left\{\coth\left(\frac{m_{s}\mu_{0}H}{kT}\right)-\frac{kT}{m_{s}\mu_{0}H}\right\}$$

where c is the fraction of SPIONs in the sampling volume, $m_{s}$ is the magnetic nanoparticle (MNP) saturation magnetization, $\mu_{0}$ is the vacuum permeability, H is the amplitude of the applied field, k is the Boltzmann constant, and T is the absolute temperature. When subjected to an AC magnetic field, magnetic nanoparticles typically exhibit relaxation. However, according to previous research, this relaxation becomes negligible when the frequency of the external magnetic field is low and the size of the magnetic nanoparticles is small [38,39]. A sinusoidal AC magnetic field of $H(t)=H_{0}\sin(\omega t)$ is applied externally, at which point the equation in Equation (1) becomes continuously differentiable. A Fourier transform is then used to decompose the signal into an odd-wave signal containing multiple frequencies,

(2) $$M=\sum_{j=1}^{n}A_{2j-1}\sin(2j-1)\omega t,n\geq 1$$

where $A_{i}$ is the magnitude of the i th harmonic of the AC magnetization intensity of the MNPs and n is the number of terms retained after the Fourier transform is transformed. The Taylor formula expansion of Equation (2) is as follows:

(3) $$M(t)=cm_{s}\left\{\frac{1}{3}\frac{m_{s}\mu_{0}H_{0}}{kT}\sin(\omega t)-\frac{1}{45}{\left(\frac{m_{s}\mu_{0}H_{0}}{kT}\sin(\omega t)\right)}^{3}+\cdots \right\}$$

A Fourier transform is performed on Equation (3), and the amplitudes of the fundamental and third harmonics of the AC magnetization response are extracted [40]:

(4) $$A_{1}=cm_{s}\left\{\frac{1}{3}\frac{m_{s}\mu_{0}H_{0}}{kT}-\frac{1}{60}{\left(\frac{m_{s}\mu_{0}H_{0}}{kT}\right)}^{3}+\frac{1}{756}{\left(\frac{m_{s}\mu_{0}H_{0}}{kT}\right)}^{5}+\cdots \right\}$$

(5) $$A_{3}=cm_{s}\left\{\frac{1}{180}{\left(\frac{m_{s}\mu_{0}H_{0}}{kT}\right)}^{3}-\frac{1}{1512}{\left(\frac{m_{s}\mu_{0}H_{0}}{kT}\right)}^{5}+\cdots \right\}$$

Equations (4) and (5) indicate that the amplitude of the AC magnetization response is proportional to the particle concentration, and the other parameters remain constant once the magnetic nanoparticles themselves are determined. The harmonic ratio analysis is derived by combining Equations (4) and (5):

(6) $$\frac{A_{1}}{A_{3}}=60{\left(\frac{kT}{m_{s}\mu_{0}H_{0}}\right)}^{2}-3+\cdots$$

In Equation (6), the harmonic amplitude ratio is functionally related only to temperature. Thus, the relationship between the harmonic ratio of the magnetization response of magnetic nanoparticles and temperature can be established. By utilizing the harmonic ratio, the process of temperature information acquisition is effectively transformed into the extraction of harmonic amplitude data. The expressions of the fundamental and third harmonic amplitudes reveal that the amplitude of the AC magnetization response decreases as the harmonic amplitude increases with temperature. Therefore, a monotonic, one-to-one correspondence exists between the temperature and the harmonic amplitude, effectively transforming the harmonic amplitude problem into a functional relationship. First, we construct a system model of the magnetic nanoparticles to define the physical problem via simulation software. Second, the material properties and relevant parameters are established, and mesh dissection is performed. The main steps of the model-building process are as follows:

(1) Definition and geometric modeling of physical problems

**Figure 1:**
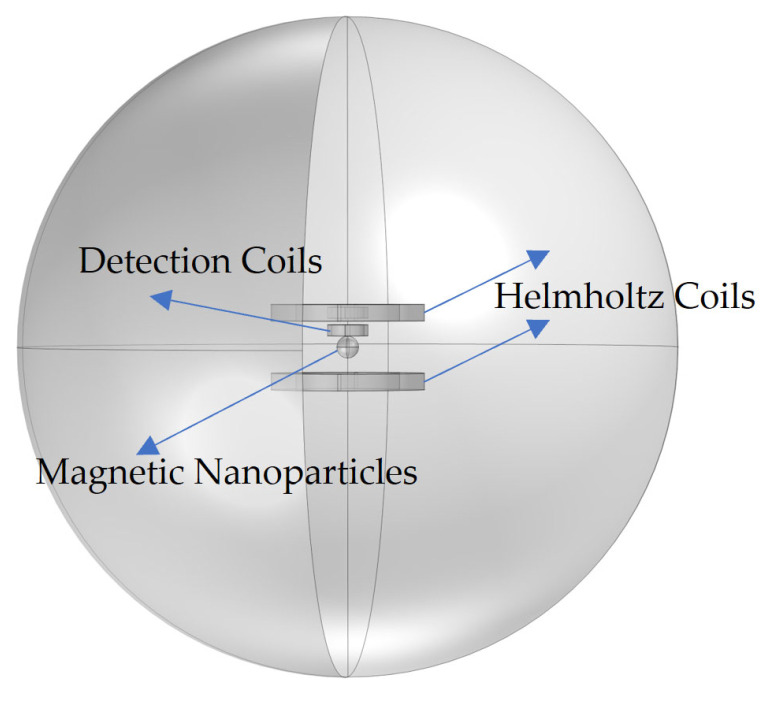
Components of the magnetic nanoparticle model.

The model is designed to effectively extract the harmonic amplitude generated by the AC magnetization response of the magnetic nanoparticles (Figure 1). The large sphere represents the region through which the magnetic field generated by the excitation coil passes, and a sufficiently large air domain is established to ensure the effective attenuation of the magnetic field. In addition, a Helmholtz coil pair is modeled in the simulation software to generate a uniform AC magnetic field. The model is centered on the magnetic nanoparticle and incorporates excitation and detection coils. The small sphere at the center represents the magnetic nanoparticle, with the Helmholtz coil providing magnetic field excitation. The detection coils are configured as differential coils to eliminate external magnetic field interference, ensuring accurate final results.

(2) Define material properties and related parameters

Before the simulation, the magnetization properties of the magnetic nanoparticles used in the magnetization model system are defined, including the relevant parameters and details of the sizes and positions of the nanoparticles. The inner diameter, outer diameter, height, wire diameter, and number of turns of the excitation coil are also defined. The detailed parameters are shown in Table 1 and Table 2.

(3) Mesh sectioning

**Figure 2:**
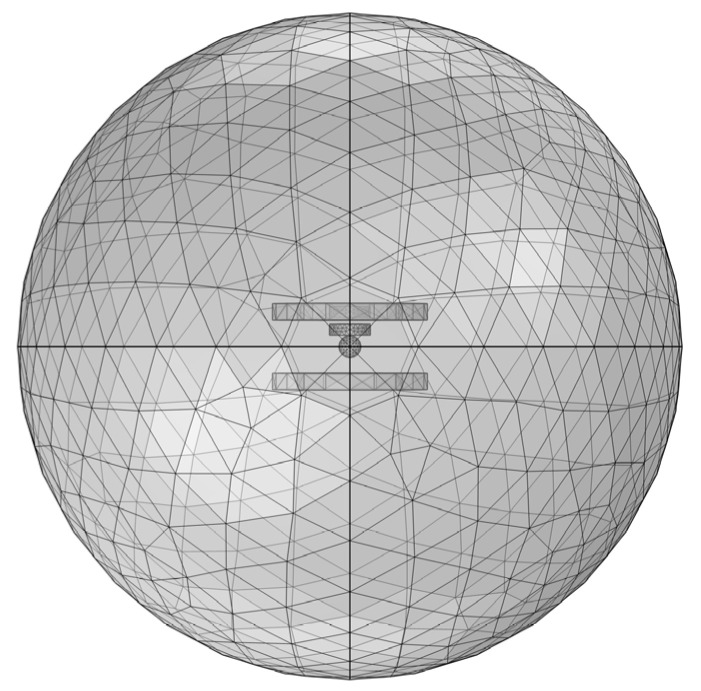
Grid sections of the magnetic nanoparticle model.

Mesh profiling is applied to magnetic nanoparticles, coils, and the surrounding air domain. Owing to the small size of the nanoparticles, a fine mesh is used, with a minimum cell size of 2.4 mm. The coil is meshed with a minimum cell size of 6 mm, while a regular mesh size is applied to the air domain. The final mesh results are shown in Figure 2.

## 3. Methods

After the entire magnetic nanoparticle system was modeled, it was validated under different conditions of magnetic field excitation.

### 3.1. Excitation in Different Magnetic Fields

#### 3.1.1. Low-Frequency AC Magnetic Field Excitation

**Figure 3:**
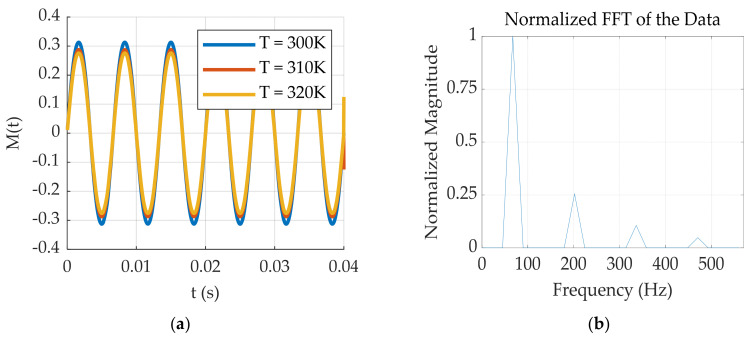
(**a**) Magnetization response at different temperatures; (**b**) normalized spectrum of the magnetization response.

The AC magnetization strength of magnetic nanoparticles decreases with increasing temperature. The magnetization response and Fourier transform of the nanoparticles at different temperatures are shown in Figure 3.

The magnetization response at 320 K, shown in Figure 3a, is Fourier transformed, and its magnetization spectrum is normalized, resulting in Figure 3b. Owing to the nonlinear nature of the Langevin function, the magnetization intensity no longer exhibits a single-frequency response but consists of multiple odd harmonics.

**Figure 4:**
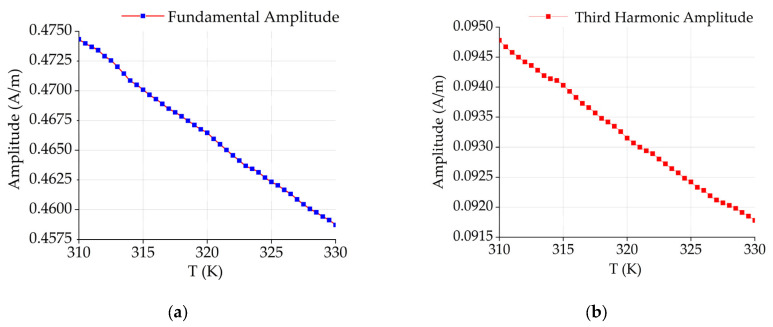
(**a**) Trend of the fundamental wave with temperature; (**b**) trend of the third harmonic with temperature.

The Fourier transform of the AC magnetization response is performed separately at different temperatures, and the fundamental and third harmonic values are extracted. The fundamental and third harmonics of the AC magnetization response of the magnetic nanoparticles gradually decrease as the temperature increases (Figure 4). This monotonic relationship between the temperature and the harmonic amplitude can be used to acquire temperature information.

The AC magnetization response of magnetic nanoparticles under low-frequency AC magnetic field excitation contains only odd harmonic components, and the number and amplitude of these harmonics affect the accuracy of the temperature measurement. To improve the accuracy, a magnetic field excitation model for the AC‒DC superposition state is constructed, generating even harmonics in the MNPs and increasing both the number and amplitude of the harmonic components.

#### 3.1.2. AC‒DC Superposition Magnetic Field Excitation

**Figure 5:**
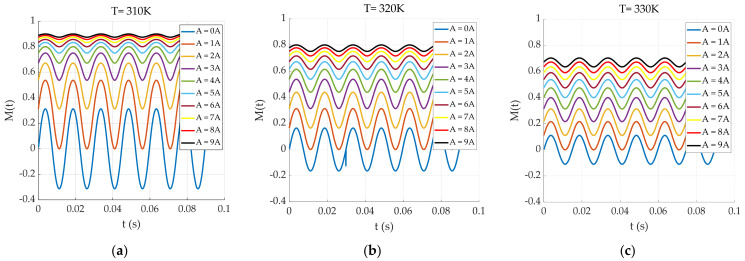
(**a**) Magnetization response of the particles at T = 310 K; (**b**) magnetization response of the particles at T = 320 K; (**c**) magnetization response of the particles at T = 330 K.

The magnetization curve of magnetic nanoparticles can be divided into saturated regions and unsaturated regions. When a DC magnetic field is superimposed on an AC magnetic field, the AC field becomes biased. When the externally applied DC magnetic field is small, the magnetic nanoparticles remain unsaturated, and their magnetization response increases rapidly. However, when the applied DC bias field is larger, the magnetization curve of the nanoparticles enters the saturation region, and the further superimposition of a magnetic field does not cause significant changes in the magnetization response. Therefore, in studies of temperature measurement under AC‒DC superposition magnetic field excitation, the DC bias field must be carefully controlled to maintain the magnetization response of the nanoparticles in the unsaturated region. Under AC‒DC superimposed excitation, the magnetization response spectrum of the magnetic nanoparticles contains both odd and even harmonics, and in this case, Equation (1) becomes

(7) $$M(t)=cm_{s}\left\{\coth\left(\frac{m_{s}\mu_{0}\left(H_{dc}\sin(\omega t)+H_{ac}\right)}{kT}\right)-\frac{kT}{m_{s}\mu_{0}\left(H_{dc}\sin(\omega t)+H_{ac}\right)}\right\}$$

where $H_{dc}$ is the magnitude of the DC magnetic field and $H_{ac}$ is the magnitude of the AC magnetic field. The harmonic amplitude measurement model for the AC‒DC superposition state is analyzed, and the magnetization response of the particles is shown in Figure 5.

**Figure 6:**
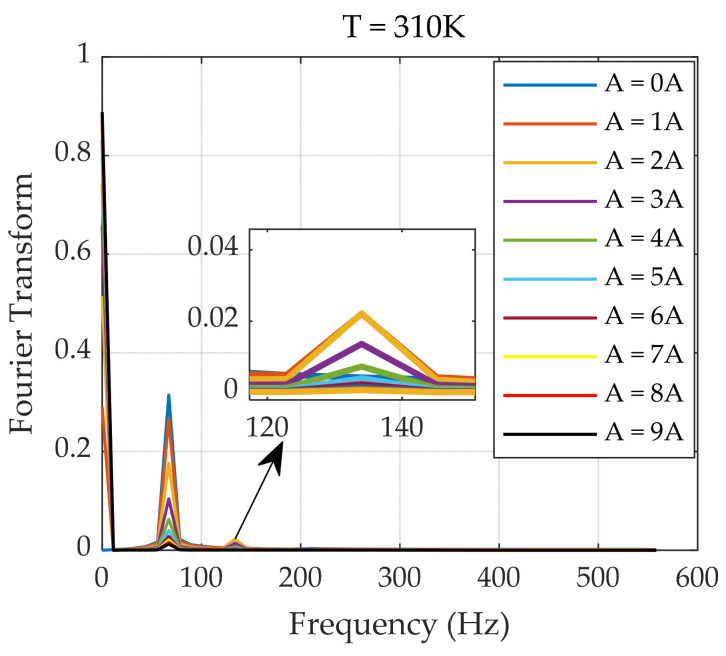
Plots of the Fourier analysis of the magnetization response under an AC‒DC superposition magnetic field.

Figure 5 shows the magnetization response curves of the magnetic nanoparticles under different AC‒DC field superposition states, where the value of A represents the magnitude of the applied DC bias magnetic field. With the gradual increase in the applied DC magnetic field, the magnetization response curve of the nanoparticles shifts upward, and the particle magnetization response approaches a constant value. Therefore, the control and selection of the DC-bias magnetic field are essential when the AC‒DC superposition field is used for coexcitation. The Fourier transform is applied to the magnetization response under AC‒DC superposition at 310 K, and the results are shown in Figure 6.

With the increase in the DC bias magnetic field, as the magnetization response approaches a constant value, its Fourier transform shows that there is an impulse excitation function at zero frequency. Additionally, stable even harmonics are generated according to the Fourier transform. Figure 6 also shows that as the harmonic order increases, the amplitudes of the components obtained from the Fourier transform decrease, and noise perturbations further affect the final result. Therefore, an analysis of the harmonic components is necessary during the harmonic acquisition process. The fundamental to sixth harmonics extracted under different applied DC bias magnetic fields are shown below.

**Figure 7:**
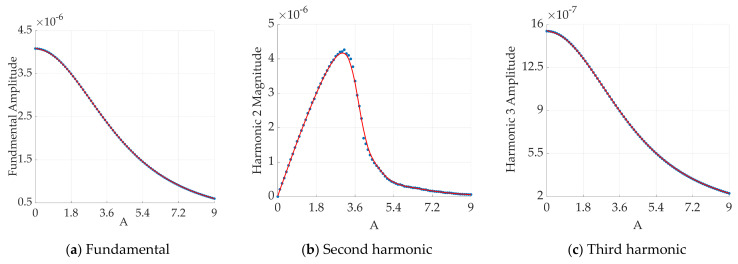

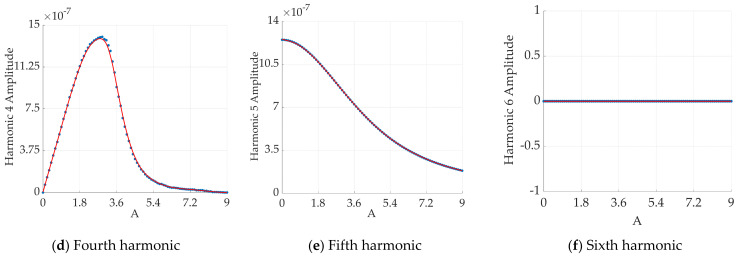
Fourier transform harmonic plots of the magnetization response with increasing DC magnetic field (**a**–**f**).

The blue data points in Figure 7 represent the harmonic values obtained at different applied bias magnetic fields, and the red line represents the fitted curve. As shown in Figure 7, with the continuous increase in the DC magnetic field, the value of the odd harmonic component consistently decreases, eventually approaching zero. In contrast, the even harmonic component first increases and then decreases with an increasing DC magnetic field. Since the sixth harmonic is a higher-order harmonic, it is difficult to extract its amplitude effectively; thus, the role of the sixth harmonic component is disregarded. Figure 7 shows that the amplitude of the second harmonic becomes larger than that of the third harmonic as the DC magnetic field increases. Therefore, superimposing AC‒DC magnetic field excitations can increase the accuracy of temperature measurement.

**Figure 8:**
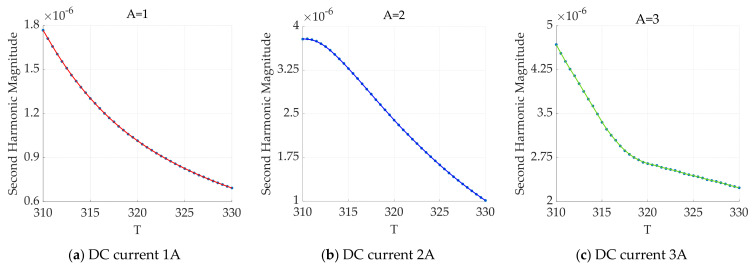
Schematic diagram of the variation in the second-harmonic amplitude with temperature under different DC magnetic field superpositions (**a**–**c**).

The variation in the second-harmonic amplitude with temperature under different applied DC magnetic fields is shown in Figure 8.

As shown in Figure 8, the amplitude of the second harmonic gradually decreases with increasing temperature and DC bias magnetic field. Under an increasing DC bias magnetic field, the magnetization response of the magnetic nanoparticles is rich in harmonic information, increasing the accuracy of harmonic amplitude acquisition. However, an analysis of the results from the superposition of AC–DC magnetic fields and the low-frequency AC magnetic field reveals that only the even-order harmonic components increase, and multiple-frequency combination components do not form. To further enrich the harmonic distribution across different frequencies, a dual-frequency superimposed magnetic field excitation model in the AC–AC superposition state is constructed.

#### 3.1.3. Dual-Frequency Superposition Magnetic Field Excitation

**Figure 9:**
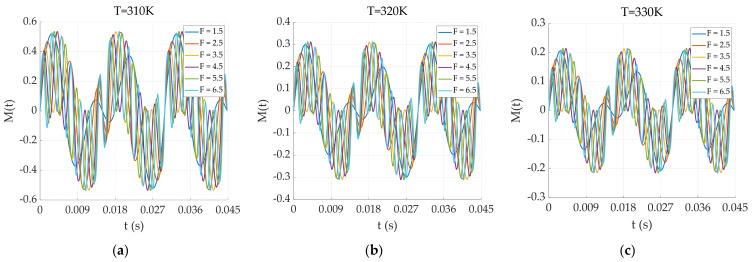
Magnetization responses under the dual-frequency superposition magnetic field excitation of the particles at (**a**) T = 310 K; (**b**) T = 320 K; (**c**) T = 330 K.

By externally applying dual-frequency superposition magnetic field excitation, the magnetization response of the particles generates a mixed-frequency signal, such as at $f_{1}\pm 2f_{2}$ and $2f_{1}\pm f_{2}$ Hz. The frequencies of $f_{1}$ and $f_{2}$ are effectively controlled, allowing richer harmonic amplitude information to be obtained at lower frequencies, thereby increasing the accuracy of harmonic amplitude acquisition and ultimately enhancing the precision of temperature information extraction. Dual-frequency superposition excitation involves applying two different AC signal frequencies to the magnetic nanoparticles during the magnetization process. Under dual-frequency superposition excitation, Equation (1) is modified as follows:

(8) $$M(t)=cm_{s}\left\{\coth\left(\frac{m_{s}\mu_{0}H_{0}\left(\sin\left(\omega_{1}t\right)+\sin\left(\omega_{2}t\right)\right)}{kT}\right)-\frac{kT}{m_{s}\mu_{0}H_{0}\left(\sin\left(\omega_{1}t\right)+\sin\left(\omega_{2}t\right)\right)}\right\}$$

where $\omega_{1}$ and $\omega_{2}$ represent the different angular frequencies of the two excitation fields and $H_{0}$ represents the dual-frequency excitation field amplitude. In this case, the definition of $\omega_{2}=F\times \omega_{1}$, equivalent to $f_{2}=F\times f_{1}$. We established a base frequency and a corresponding multiplier frequency derived from the base frequency, defining the multiplier as F. Both sides of the formula represent frequencies, and F is the multiplier parameter. Therefore, in the following figures, F is unitless. The results of the simulation using the two excitations at different frequencies are shown in Figure 9.

**Figure 10:**
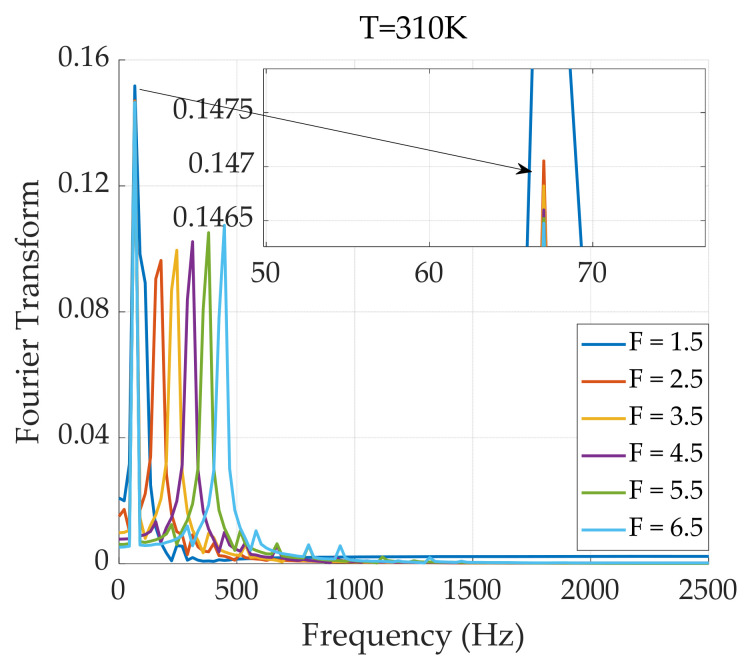
Fourier transform spectrum of the magnetization response of a particle excited by a 310 K dual-frequency superimposed magnetic field.

In Figure 9, the factor F represents the F multiplicity of one magnetic field frequency for the other magnetic field frequency in the dual-frequency superposition state. A Fourier analysis is performed on the magnetization response of the 310 K particles in Figure 9, and the results are shown in Figure 10.

As shown in Figure 10, under dual-frequency superimposed magnetic field excitation, the relaxation of magnetic nanoparticles increases as the excitation frequency continues to rise. This alters the AC magnetization response of the particles, leading to greater energy dissipation, which reduces both the value and accuracy of the extracted harmonics of the particle’s magnetization response. Therefore, the frequency factor must be carefully considered.

### 3.2. Error Analysis

The factors of truncation error, excitation frequency, and excitation magnetic field amplitude affect the accuracy of amplitude information acquisition, which affects the accuracy of temperature information acquisition. To further increase the accuracy of temperature information acquisition, a corresponding study of these factors is conducted in this subsection.

#### 3.2.1. Effect of Truncation Error

For accurate temperature information acquisition in the presence of external magnetic field excitation, a Taylor series expansion of the magnetization response model is necessary. Since the Langevin function is nonlinear, its Taylor expansion includes residual terms. Increasing the number of terms retained in the expansion of the Langevin function increases the accuracy of the final temperature information. However, when high-order harmonics of the magnetization response are selected for analysis, their harmonic amplitude components are small, making them difficult to extract efficiently. Additionally, when the selected harmonic amplitude component is small, the associated error becomes significant, reducing the overall accuracy. Therefore, a comprehensive analysis of the number of terms in the Taylor expansion of the Langevin function is needed. Although the applied external excitation magnetic fields may differ, their amplitude expressions include ${\left(\mu_{0}m_{S}H/kT\right)}^{2n-1}$ terms. When n is assigned different values, the amplitude expansion equation of the magnetization response has a different number of terms. Thus, the ${\left(\mu_{0}m_{S}H/kT\right)}^{2n-1}$ parameters with different terms are defined as the n th-order truncation.

**Figure 11:**
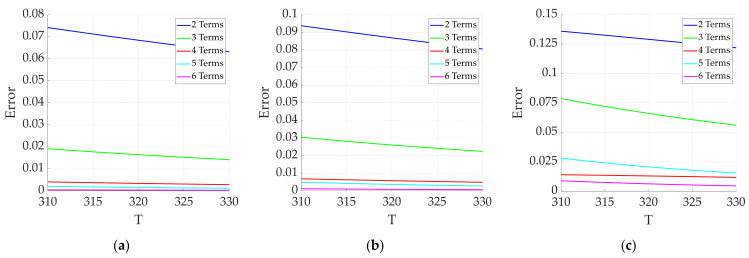
Errors between different expansion orders and the original function under (**a**) low-frequency AC magnetic field excitation; (**b**) AC–DC superposition magnetic field excitation; (**c**) dual-frequency superposition magnetic field excitation.

The magnetization responses of magnetic nanoparticles under different excitation magnetic fields are expanded according to the second-order to sixth-order truncations, and the results are shown in Figure 11.

Figure 11 presents an analysis of the magnetization response under three different excitation magnetic fields at various expansion orders. Different values of n represent different orders of expansion, and the error values indicate the deviations from the original function when different numbers of terms are selected. As shown in Figure 11, as the number of selected orders increases, the error value gradually decreases. The errors from the second-order and third-order truncations are significantly larger than those from the higher-order terms. When the expansion terms reach the fifth or sixth order, the errors are relatively small. However, selecting a sixth-order expansion term reduces the temporal resolution during temperature information extraction. Therefore, the fifth-order model is chosen for further analysis.

#### 3.2.2. Effect of the Excitation Frequency

When the excitation frequency is too high, the dynamic magnetization response of the nanoparticles no longer satisfies Equation (1) because of relaxation. Conversely, if the frequency is too low, the bandwidth difference between the magnetization responses of the particles decreases. This makes it challenging to distinguish between the amplitudes of the fundamental wave and the third harmonic, thereby reducing the accuracy of harmonic amplitude extraction. Under AC magnetic field excitation, the power lost by the particles due to this relaxation effect is referred to as dissipated power, and it is expressed as

(9) $$p=\pi f\mu_{0}\mu^{\prime \prime }H_{0}^{2}$$

where f is the frequency of the alternating magnetic field, $\mu_{0}$ is the vacuum permeability, $\mu^{\prime \prime }$ is the imaginary part of the AC magnetization, and $H_{0}$ is the amplitude of the excitation field. Equation (9) shows that the magnitude of the dissipated power is proportional to the frequency of the AC magnetic field. Usually, the frequency range of the excitation magnetic field can be chosen to take values between tens and hundreds of Hz, in which case the power dissipation of the magnetic nanoparticles can be neglected.

#### 3.2.3. Effect of Excitation Magnetic Field Amplitude

According to the mathematical expressions, when expanding the function, it is necessary to ensure that the dimensionless parameter argument in the function is much less than 1: $\left(\mu_{0}m_{S}H/kT\right)$<< 1. When the magnetic nanoparticles themselves are determined, $m_{s}$, $\mu_{0}$, k, and T in $\left(\mu_{0}m_{S}H/kT\right)$ are all fixed values, and the only variable is H.

When the amplitude of the excitation magnetic field is too small, the magnetization response of the magnetic nanoparticles decreases, resulting in a lower signal-to-noise ratio. Conversely, when the amplitude of the excitation magnetic field is too large, the Langevin function no longer satisfies the conditions for Taylor expansion at the zero point. Therefore, the selection of the excitation magnetic field amplitude is essential. Under low-frequency AC magnetic field excitation, we analyze the error between the original Langevin function and its expansion formula under different excitation field amplitudes.

**Figure 12:**
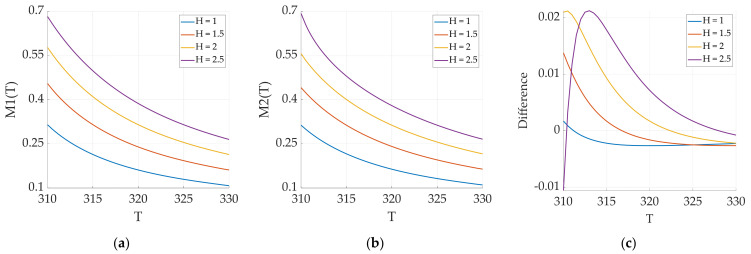
(**a**) Langevin function magnetization response; (**b**) magnetization response for the expansion of the Langevin function; (**c**) error between the Langevin original function and the expansion with the variation in the excitation magnetic field amplitude.

In Figure 12, H represents the amplitude multiplication relationship between the magnetic field and the original excitation magnetic field. Analysis of the results indicates that an increase in the magnetic field amplitude increases the magnetization response of the magnetic nanoparticles to some extent. It also increases the error between the original function and the expanded function. This error gradually decreases as the temperature increases since the overall magnetization response of the particles decreases with increasing temperature. This change in the error values indicates the need for the dynamic selection of the magnetic field strength in practical applications.

## 4. Results

### 4.1. System Simulation

Models for low-frequency AC magnetic field excitation, AC–DC superposition magnetic field excitation, and dual-frequency superposition magnetic field excitation have been effectively constructed and analyzed. The factors influencing the error have also been investigated. As an example, this section describes the analysis of the results in the simulation software at t = 0.01 s under low-frequency AC magnetic field excitation.

**Figure 13:**
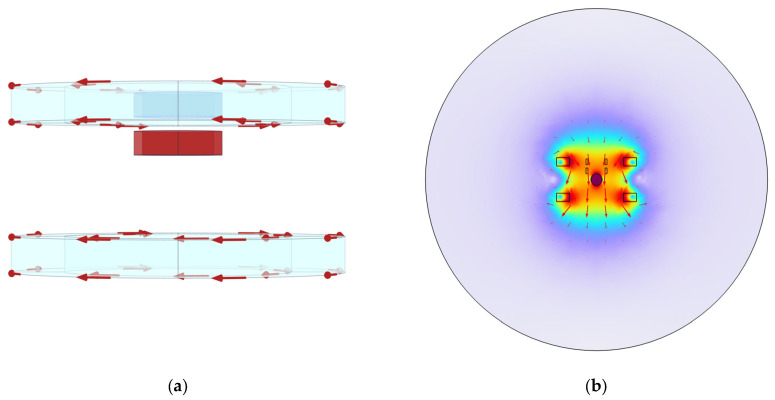
(**a**) Direction of the current under the transient field; (**b**) magnetic flux density along the *Y*-axis under the transient field.

First, “Coil Geometry Analysis” is added, and after the physical field interface is selected, the study is conducted in the transient frequency domain. The current direction under low-frequency AC magnetic field excitation is analyzed, and the current direction schematic and magnetic flux density are shown in Figure 13.

**Figure 14:**
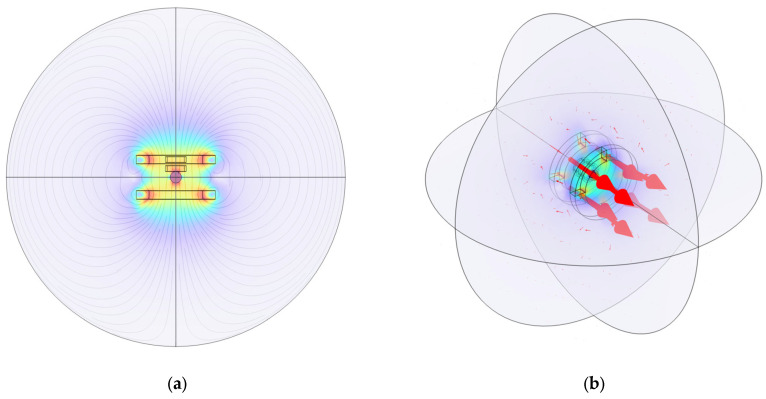
(**a**) Schematic of the magnetic field distribution; (**b**) schematic representation of the magnetic field distribution in space.

Since a Helmholtz coil is used for the analysis, it is necessary to pass an alternating current of the same direction and amplitude into the excitation coil. Figure 13a shows the current flow in the excitation coil at t = 0.01 s, and Figure 13b illustrates the formation of a uniform magnetic field at the center of the coil. At this point, the schematic distribution of the magnetic field generated by the Helmholtz coil, along with the spatial magnetic flux density in the transient field, is as shown in Figure 14.

**Figure 15:**
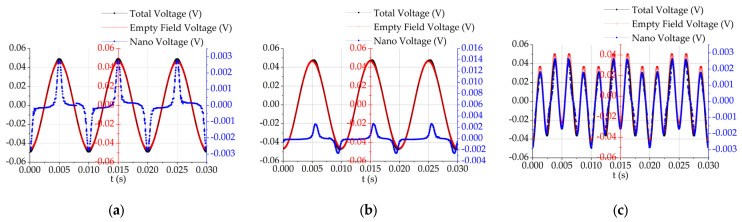
(**a**) Voltage signals under low-frequency AC magnetic field excitation; (**b**) voltage signal under AC–DC superposition magnetic field excitation; (**c**) voltage signals under dual-frequency superposition magnetic field excitation.

Owing to the presence of the detection coil pair, a voltage signal is generated in the detection coil when the external magnetic flux changes. Since the entire detection coil pair is within a dynamically changing magnetic field, the coil detects not only the induced voltage signals generated by the magnetic nanoparticles, but also the signals from the externally applied magnetic field, as it is an alternating magnetic field. The different detection voltage signals obtained through the coil under various external excitation magnetic fields are shown in Figure 15.

In Figure 15, the black line represents the total voltage signal detected by the coil, which includes both the voltage signal from the empty field and the induced voltage generated by the particles. The red curve represents the voltage signal of the empty field, which is detected without the magnetic nanoparticles in the simulation model. The blue curve represents the voltage signal generated by the magnetic nanoparticles, as detected by the differential coil. The voltage signal of the magnetic nanoparticles varies for different magnetic field excitations. Voltage changes are observed before and after the magnetic nanoparticles are placed, and this confirms that the nanoparticles generate a magnetization response under the influence of the AC magnetic field. By effectively analyzing and extracting this magnetization response signal, temperature information can be obtained.

### 4.2. Temperature Inversion Algorithm

The magneto-temperature model of the magnetic nanoparticles is constructed by obtaining the fundamental and harmonic amplitudes of the AC magnetization response under AC magnetic field excitation, and converting the temperature information into harmonic amplitude data for extraction. These harmonic amplitude data are then inverted to retrieve the corresponding temperature information. Various temperature inversion algorithms are applied to perform the inversion, enabling high-precision temperature extraction.

Since the particle swarm optimization (PSO), gray wolf optimization (GWO), and differential evolution (DE) algorithms have good generality, these three individual algorithms are used for temperature inversion. Subsequently, joint algorithms are developed on the basis of these single algorithms. Specifically, temperature inversion is performed via autonomous group particle swarm optimization (AGPSO), the opposition learning gray wolf optimizer (OLGWO), and particle swarm optimization–gray wolf optimization (PSO-GWO).

**Figure 16:**
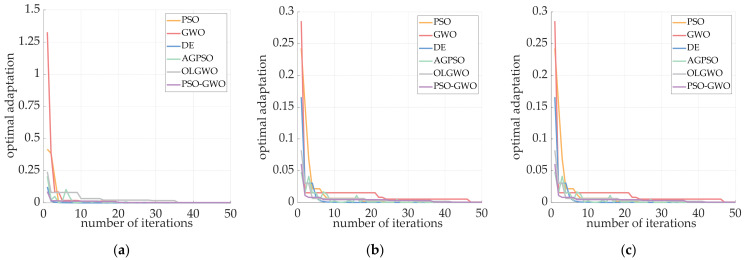
Adaptation distributions under (**a**) low-frequency AC magnetic field excitation; (**b**) AC‒DC superposition magnetic field excitation; (**c**) dual-frequency superposition magnetic field excitation.

Low-frequency AC magnetic field excitation, AC–DC superposition magnetic field excitation, and dual-frequency superposition magnetic field excitation are applied externally. Temperature inversion of the magnetization response of the magnetic nanoparticles under low-frequency magnetic field excitation is performed via the six aforementioned algorithms. The distribution of the optimal fitness values is shown in Figure 16.

Figure 16a shows that under low-frequency AC magnetic field excitation, the adaptation of the single algorithm is much faster than that of the combined optimized algorithm. In Figure 16b, the number of iterations required to reach the optimal fitness under AC–DC superposition magnetic field excitation is much greater than that under low-frequency AC magnetic field excitation. In Figure 16c, as the excitation magnetic field becomes more complex, the iterative adaptation becomes chaotic, and the number of iterations increases. The fitness distribution graph shows that the fitness distribution of magnetic nanoparticles under inversion using the joint algorithm is superior to that of the single algorithm, but the number of inversion iterations is slightly greater with the integrated algorithm.

### 4.3. Data Acquisition and Inversion Results

**Figure 17:**
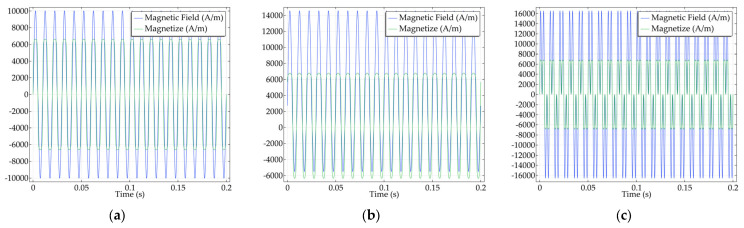
(**a**) Low-frequency AC magnetic field excitation and particle magnetization response; (**b**) AC–DC superposition magnetic field excitation and particle magnetization response; (**c**) dual-frequency superposition magnetic field excitation and particle magnetization response.

The system simulation of magnetic nanoparticles is performed under low-frequency AC magnetic field excitation, AC‒DC superposition magnetic field excitation, and dual-frequency superposition magnetic field excitation, and the results are shown in Figure 17 below.

Figure 17a–c show the magnetization responses of the magnetic nanoparticles under different magnetic field excitations, as well as the distributions of the excitation magnetic fields. As shown in Figure 17b, with the superposition of the DC bias field, the excitation magnetic field clearly shows an overall upward shift, and the response of the particles is partially distorted. Figure 17c shows that both the magnetic field excitation and the magnetization response of the magnetic nanoparticles in the dual-frequency state change rapidly. The harmonic amplitudes of the magnetization responses under different magnetic fields are extracted, and temperature inversion is performed via the respective inversion algorithms. The error values of the temperature inversion and the time required for each are presented in Table 3.

**Figure 18:**
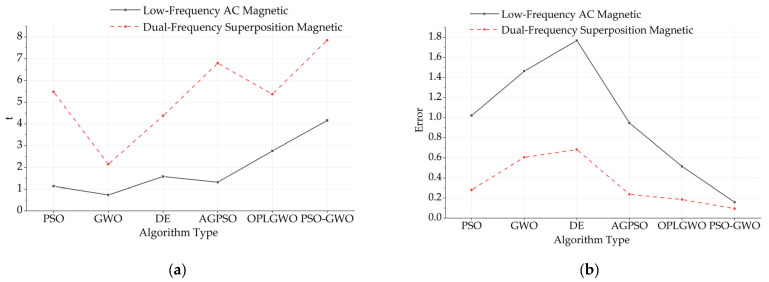
(**a**) Comparison of temperature inversion times for different excitation magnetic fields; (**b**) comparison of temperature inversion errors for different excitation magnetic fields.

By analyzing the data in the table, it is evident that when different algorithms are applied to the same magnetic field, the joint algorithm yields higher accuracy but takes more time than the single algorithm does. Under the same algorithm, the error is smaller with dual-frequency superposition magnetic field excitation than with low-frequency or AC–DC superposition, although the required inversion time is correspondingly longer. Additionally, the magnetic field in the AC–DC superposition state is less accurate than that in the dual-frequency superposition state, and its real-time performance is worse than that of the low-frequency AC magnetic field. Therefore, further analysis of the magnetic field in the AC‒DC superposition state is not conducted. The table is analyzed, and the results are shown in Figure 18.

As shown in Figure 18, when real-time performance is prioritized over accuracy, the AGPSO algorithm can be effectively used for temperature inversion under low-frequency AC magnetic field excitation. It provides better real-time performance while maintaining a low error and reducing the time required by 52% and 68% compared with those of the OLGWO and the PSO-GWO, respectively. Conversely, when accuracy is prioritized over real-time performance, the PSO-GWO algorithm can be used for temperature inversion under dual-frequency superposition magnetic field excitation, reducing the final temperature inversion error to 0.094 K.

## 5. Discussion

In this study, we investigate the magnetization responses of magnetic nanoparticles under three different types of magnetic field excitation: low-frequency AC, AC‒DC superposition, and dual-frequency superposition. The nanoparticle response is subsequently converted into temperature data through the application of various inversion algorithms that leverage their temperature-sensitive properties.

We begin by conducting a spectral analysis of the magnetization response under low-frequency AC magnetic field excitation, observing that only odd harmonics are present in this low-frequency state. Building on this observation, we develop the AC–DC superposition magnetic field excitation model and the dual-frequency superposition magnetic field excitation model. The magnetization response spectrum under AC–DC superposition excitation includes both odd and even harmonics, while the spectrum under dual-frequency superposition excitation exhibits additional combined frequency components.

Subsequently, we analyze the factors influencing the temperature measurement of magnetic nanoparticles and optimize their temperature measurement capabilities by examining their intrinsic properties. Additionally, the behavior of the magnetic nanoparticle system is modeled using simulation software, which simulates its response under various excitation states. Magnetization response signals for each state are obtained separately, and the resulting data are processed to extract information.

Finally, the temperature inversion of magnetic nanoparticle amplitude information is performed effectively, utilizing different temperature inversion algorithms to validate and complement one another.

We employed six different inversion algorithms, comprising three basic algorithms and three optimized algorithms developed through improvements on the basic ones. The temperature inversion process aims to achieve both accuracy and real-time performance. For each algorithm, we performed parameter selection and optimization to ensure accuracy and efficiency. Additionally, we conducted a best-fitness analysis and compared the final results. Ultimately, we found that, under low-frequency AC magnetic field excitation, the AGPSO algorithm demonstrated a significant improvement in real-time performance, reducing the time required by 52% and 68% compared with that of the other two joint algorithms. Under dual-frequency superposition magnetic field excitation, the PSO-GWO algorithm effectively reduced the temperature inversion error from 0.158 K to 0.094 K.

In the future, further investigations are needed regarding the strategy of using magnetic nanoparticles for high-precision temperature measurement while reducing the time required for the measurement process to improve real-time performance. Additionally, experimental validation of the proposed methods for temperature measurement is necessary.

## 6. Conclusions

This study investigates a method for achieving high-precision and real-time temperature acquisition in micro and nano environments. Leveraging the temperature-sensitive properties of magnetic nanoparticles, the study explores the use of their magnetization response to effectively acquire temperature information. Three excitation models—low-frequency AC, AC–DC superposition, and dual-frequency superposition—are proposed, along with corresponding inversion algorithms. These models and algorithms are optimized to enhance both the accuracy and real-time performance of temperature measurement. The results indicate that the AGPSO algorithm significantly improves the accuracy of temperature acquisition under low-frequency AC magnetic field excitation, while the PSO-GWO algorithm notably reduces temperature measurement errors under dual-frequency superposition magnetic field excitation.

## Figures and Tables

**Table 1:** Design of parameters related to excitation and detection coils.

	Number of Turns	Inner Diameter	Outer Diameter	Height
excitation coil	140	47.5 mm	70 mm	15 mm
detection coils	62	15 mm	18.5 mm	10 mm

**Table 2:** Design of parameters related to particle magnetization model.

MNPs Diameter	Boltzmann Constant	Air Conductivity
30 nm	$1.38\times 10^{-23}$	0.001 S/m
saturation magnetization	simulation model radius	airspace radius
7000 A/m	10 mm	300 mm
cross-sectional area of the conductor	relative permeability	relative dielectric constant
$1\times 10^{-6}$ m^2^	1	1

**Table 3:** Distribution of temperature inversion error values and real-time tables.

Δ(k)/t(s)	Low-Frequency AC	AC–DC	Dual-Frequency
PSO	1.021/1.132	0.638/2.037	0.281/5.470
GWO	1.462/0.735	1.037/1.412	0.605/2.146
DE	1.768/1.582	0.603/2.145	0.683/4.368
AGPSO	0.947/1.318	0.394/4.741	0.237/6.793
OPLGWO	0.515/2.756	0.291/3.149	0.185/5.361
PSO-GWO	0.158/4.159	0.431/3.571	0.094/7.846

## Data Availability

The simulation model can be shared upon reasonable request to the authors.
